# Molecular Detection of Canine Distemper Virus in Portugal: What Explains the Post-2020 Decline? A Retrospective RT-qPCR Study

**DOI:** 10.3390/v18070734

**Published:** 2026-07-02

**Authors:** Ricardo Lopes, Cristina Costa Santos, Hugo Lima de Carvalho, Filipe Sampaio, Cátia Fernandes, Andreia Garcês, Carlos Sousa, Ana Rita Silva, Hugo Silva, Luís Cardoso, Nuno Alegria, Elsa Leclerc Duarte, Ana Cláudia Coelho

**Affiliations:** 1 CEDIVET Veterinary Laboratories, Lionesa Business Hub, R. Lionesa 446 C24, 4465-671 Leça do Balio, Portugal; lopes.rmv@gmail.com (R.L.); hugo.carvalho@cedivet.pt (H.L.d.C.); filipe.sampaio@cedivet.pt (F.S.); 2 Department of Veterinary Sciences, University of Trás-os-Montes e Alto Douro (UTAD), 5000-801 Vila Real, Portugal; nalegria@utad.pt (N.A.); accoelho@utad.pt (A.C.C.); 3 Department of Veterinary and Animal Sciences, University Institute of Health Sciences (IUCS), CESPU, 4585-116 Gandra, Portugal; 4 UNIPRO—Oral Pathology and Rehabilitation Research Unit, University Institute of Health Sciences (IUCS), CESPU, 4585-116 Gandra, Portugal; 5 Molecular Diagnostics Laboratory, Unilabs Portugal, Lionesa Business Hub, R. Lionesa, 4465-671 Leça do Balio, Portugal; hugo.silva@unilabs.com; 6 RISE-Health, Department of Community Medicine, Information and Health Decision Sciences, Faculty of Medicine, University of Porto, Alameda Prof. Hernâni Monteiro, 4200-319 Porto, Portugal; csantos@med.up.pt; 7 Cytology and Hematology Diagnostic Services, Laboratory of Histology and Embryology, Department of Microscopy, ICBAS-School of Medicine and Biomedical Sciences, University of Porto, Rua de Jorge Viterbo Ferreira, 228, 4050-313 Porto, Portugal; 8 AniCura Santa Marinha Veterinary Hospital, R. Dom Henrique de Cernache 183, 4400-625 Vila Nova de Gaia, Portugal; catia.fernandes@anicura.pt; 9 Wildlife Rehabilitation Centre (CRAS), Veterinary Teaching Hospital, University of Trás-os-Montes e Alto Douro (UTAD), 5000-801 Vila Real, Portugal; andreiamvg@gmail.com; 10 Animal and Veterinary Research Centre (CECAV), Associate Laboratory for Animal and Veterinary Sciences (AL4AnimalS), University of Trás-os-Montes e Alto Douro (UTAD), 5000-801 Vila Real, Portugal; 11 Molecular Pathology Laboratory, SYNLAB Portugal, Av. Columbano Bordalo Pinheiro, 75A, 1070-061 Lisboa, Portugal; carlos.sousa@synlab.pt (C.S.); ana-rita.silva@synlab.pt (A.R.S.); 12 PerMed Research Group, RISE-Health, Faculty of Medicine, University of Porto, 4200-319 Porto, Portugal; 13 Department of Veterinary Medicine, School of Science and Technology, University of Évora, Polo da Mitra, Apartado 94, 7002-554 Évora, Portugal; emld@uevora.pt; 14 Mediterranean Institute for Agriculture, Environment and Development (MED), Global Change and Sustainability Institute (CHANGE), University of Évora, Polo da Mitra, Apartado 94, 7002-554 Évora, Portugal

**Keywords:** canine distemper virus, dogs, laboratory surveillance, *Morbillivirus canis*, One Health, Portugal, RT-qPCR

## Abstract

Canine distemper virus (CDV), currently classified within the species *Morbillivirus canis*, is a major vaccine-preventable pathogen of domestic dogs and a wide range of susceptible wildlife species. Still, laboratory-confirmed epidemiological data from Portugal remain scarce. This retrospective study investigated CDV molecular detection in 637 diagnostic samples from dogs with clinical suspicion of canine distemper, received from 190 veterinary medical centres across Portugal between 2013 and 2025. Cerebrospinal fluid, EDTA-anticoagulated whole blood, rectal swabs, and conjunctival swabs were tested for CDV RNA using a reverse transcription real-time PCR (RT-qPCR) assay in a qualitative approach. Overall, 215 submissions were CDV RT-qPCR-positive, corresponding to a positivity of 33.8% (95% confidence interval: 30.2–37.5%). Positivity was not significantly associated with sex, age, or Nomenclature of Territorial Units for Statistics level 2 (NUTS 2) region, but differed significantly according to specimen type, with the highest detection observed in EDTA-anticoagulated whole blood and conjunctival swabs. Mixed-breed dogs were over-represented among submitted samples and positive cases, probably reflecting management, exposure, and vaccination-related factors rather than intrinsic breed susceptibility. The central finding was a pronounced post-2020 decline in CDV RT-qPCR positivity, with very low or absent annual detection between 2021 and 2025. This pattern indicates reduced molecular detection within a passive diagnostic population but should not be interpreted as evidence of national elimination. Continued vaccination and strengthened surveillance at the domestic dog–wildlife interface remain essential.

## 1. Introduction

Canine distemper virus (CDV), currently classified within the species *Morbillivirus canis*, is an enveloped, non-segmented, negative-sense RNA virus belonging to the genus *Morbillivirus*, subfamily *Orthoparamyxovirinae*, family *Paramyxoviridae*, order *Mononegavirales*. It remains one of the most important viral pathogens of domestic dogs and susceptible wildlife [1]. Contemporary phylogenetic studies support the existence of marked global genetic diversity, with at least 17 major genotypes or lineages described in the literature. The haemagglutinin (H) gene remains a key marker of viral adaptation, host interaction, and lineage assignment [2]. Recent analyses further suggest positive selection at receptor-binding sites of the H protein, reinforcing the importance of ongoing molecular surveillance of circulating field strains [3,4,5].

CDV is now widely recognised as a multi-host pathogen with major clinical and conservation relevance. Domestic dogs remain a central epidemiological host. However, current evidence suggests that viral maintenance may occur within a multi-host meta-reservoir, particularly at domestic dog–wildlife interfaces where free-roaming dogs, synanthropic mesocarnivores, and susceptible wild carnivores overlap [6]. The host spectrum is broad and includes canids, mustelids, procyonids, and felids most frequently, while infection in other mammals continues to highlight the remarkable host plasticity of the virus and its relevance for One Health-oriented surveillance and conservation medicine [3,6,7].

Although vaccination has substantially contributed to the reduction and control of canine distemper where coverage is adequate, CDV is still globally distributed and continues to cause clinically important outbreaks in owned dogs, shelter populations, free-roaming dogs, and wildlife [1,6,7,8]. In Europe, recent investigations confirm ongoing circulation in multiple wildlife and domestic–wildlife interface systems, including Italy [7], Spain (Catalonia) [9], Croatia [10], and Belgium [11]. These findings underscore that distemper remains an active epidemiological and conservation problem rather than a historical disease under full control. In Italy, several lineages, including Europe/South America-1, Europe Wildlife, and Arctic-like viruses, have been reported in domestic and wild carnivores, while other European studies document sustained transmission in red foxes, badgers, and other free-ranging hosts [1,2,7,9,10,11,12,13,14].

Clinically, CDV causes a multisystemic disease involving respiratory, gastrointestinal, mucocutaneous, and nervous systems, with disease severity influenced by host age, immune status, and viral strain [15,16,17,18,19,20]. Puppies and incompletely vaccinated dogs remain overrepresented among severe cases, and persistent or delayed neurological disease is linked to viral neurotropism and demyelinating lesions of the central nervous system [18,19]. Ante-mortem diagnosis currently relies mainly on molecular detection, especially RT-qPCR, with sample selection tailored to the stage and presentation of disease [21,22,23,24,25]. Newer digital PCR approaches may improve sensitivity in low-copy samples and support more accurate case confirmation [26].

Vaccination remains the cornerstone of prevention and is still considered a core intervention for all dogs worldwide. Current World Small Animal Veterinary Association (WSAVA) guidelines emphasise repeated puppy core vaccination until at least 16 weeks of age, with several vaccine administrations during the primary vaccination course to overcome maternally derived antibody interference [8]. Antigenic divergence between vaccine strains and contemporary field viruses has renewed interest in updated vaccine design. However, current evidence suggests that incomplete vaccine coverage, lack of vaccine compliance, and domestic dog–wildlife transmission events are more important drivers of viral persistence than any proven widespread loss of vaccine protection [5,8,27].

In Portugal, the published evidence remains fragmented but is consistent with sustained circulation of CDV at the domestic dog–wildlife interface [2,12,28,29]. Serological data have documented exposure in free-ranging wolves [12] and red foxes [12,29]. Molecular characterisation has demonstrated domestic-dog-related CDV in Iberian lynxes [13]. Long-term surveillance in northern Portugal has recorded epizootic CDV activity in wolves [2], and a clinical case series described the Lisbon outbreak of 2014–2018 [28]. Taken together, these studies suggest that current Portuguese knowledge on CDV is still based mainly on wildlife surveys and local outbreak descriptions rather than on integrated national surveillance in dogs.

Given this background, the present study retrospectively analysed a national laboratory database of CDV RT-qPCR diagnostic samples from dogs in Portugal between 2013 and 2025 (*n* = 637). Available Portuguese literature on CDV circulation and detection in domestic and wild carnivores is used only to contextualise findings. This study provides a nationwide retrospective assessment of laboratory-confirmed canine distemper in Portuguese dogs, aiming to describe demographic, clinical, and spatial patterns, assess factors associated with RT-qPCR positivity, and contextualise domestic dog infections within the framework of vaccination gaps, passive laboratory surveillance, and domestic dog–wildlife interfaces.

## 2. Materials and Methods

### 2.1. Sampling, Data Collection, and Diagnostic Procedures

Diagnostic specimens from dogs with clinical suspicion of canine distemper were submitted to CEDIVET Veterinary Laboratories (Porto, Portugal) for the molecular detection of CDV. The submitted biological materials included cerebrospinal fluid (CSF), EDTA-anticoagulated whole blood, rectal swabs, and conjunctival swabs. Samples were submitted as part of routine diagnostic investigation by 190 veterinary medical centres, including first-opinion clinics and referral hospitals, across Portugal and the autonomous insular regions.

**Figure 1:**
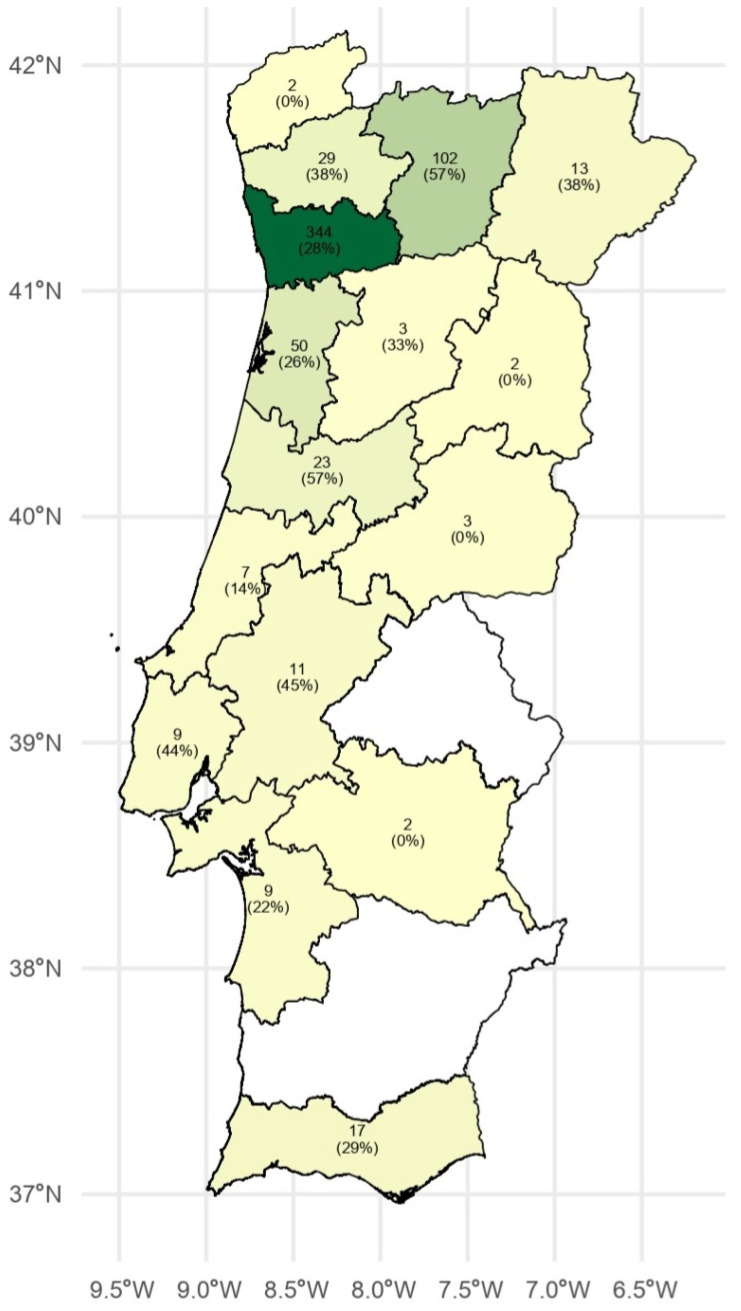
Geographical distribution of canine distemper virus RT-qPCR diagnostic submissions from clinically suspected dogs in mainland Portugal by district. Numbers indicate the absolute number of diagnostic submissions per district, and percentages in parentheses indicate district-level CDV RT-qPCR positivity, calculated as the proportion of positive samples among all valid submissions from that district. Shading reflects the number of submissions. District-level sample counts, positive counts, positivity estimates and 95% confidence intervals are provided in Table S1. Submissions from the autonomous insular regions were reported separately (*n* = 11). This passive laboratory-based distribution should not be interpreted as population-based incidence, prevalence, or regional disease risk.

Geographical distribution was mapped in R using district boundaries from the Portuguese Official Administrative Map (“Carta Administrativa Oficial de Portugal, CAOP; Direção-Geral do Território”) [30]. Diagnostic submissions were aggregated by district and displayed as absolute frequencies, with districts shaded according to the number of submissions. Submissions from the autonomous insular regions were reported separately due to their low numbers. For each district, the number of submitted samples, the number of CDV RT-qPCR-positive samples, district-level positivity and 95% confidence intervals were calculated. In Figure 1, districts are shaded according to the number of diagnostic submissions, and labels indicate the absolute number of submissions, with the corresponding CDV RT-qPCR positivity shown in parentheses. The complete district-level distribution is provided in Table S1.

All laboratory submissions recorded between 2013 and 2025 were screened for eligibility. Records were included when they referred to domestic dogs (*Canis familiaris*) submitted for CDV molecular testing based on suspected canine distemper, as indicated by the attending veterinarian, with reported absence of CDV vaccination and a valid interpretable RT-qPCR result. Records were excluded when they referred to species other than domestic dogs, invalid or inconclusive RT-qPCR results, specimen types outside the predefined categories, duplicate submissions from the same dog or sampling episode, or documented CDV vaccination incompatible with the study definition of clinically suspected unvaccinated dogs. After applying these criteria, 637 submissions were included in the final analysis.

For each diagnostic sample, the accompanied laboratory request form was reviewed to retrieve the available demographic, clinical, and epidemiological information, including breed, sex, age, vaccination and prophylactic status, clinical suspicion and/or reported clinical signs, specimen type, geographical origin, and requested diagnostic analyses. Age was analysed as a continuous variable expressed in months. Dogs lacking age information, corresponding to 15.9% of the dataset, were retained for overall analyses but excluded from age-related analyses. Breed was analysed as a binary breed group variable, comparing mixed-breed dogs with all other reported breeds combined.

### 2.2. Molecular Analysis

Viral RNA was extracted from cerebrospinal fluid, EDTA-anticoagulated whole blood, rectal swabs, and conjunctival swabs using the Promega AX9760 custom protocol on the KingFisher™ Flex system (Thermo Fisher Scientific, Waltham, MA, USA), according to the laboratory’s routine molecular diagnostic workflow. Upon receipt at the laboratory, specimens were stored at 2–8 °C until processing. When RT-qPCR was not performed immediately after extraction, RNA extracts were stored at −20 °C until analysis, and repeated freeze–thaw cycles were avoided.

Molecular detection of CDV was performed using the NZYtech Canine Distemper virus RT-qPCR Kit^®^ (NZYtech, Lisbon, Portugal), a one-step TaqMan^®^ reverse transcription real-time PCR assay designed for the qualitative in vitro detection of CDV RNA. According to the manufacturer, the primers and probe sequences show high (>95%) homology with a broad range of CDV genomes, while maintaining specificity for CDV. The assay includes CDV-specific primers and a hydrolysis probe labelled for detection in the FAM™ channel, together with an internal extraction control detected in the HEX™/JOE™/VIC™ channel.

Extracted RNA was not quantified before RT-qPCR, and no inter-sample normalisation to total RNA concentration or to a host reference gene was performed, because the study was designed to evaluate qualitative CDV RT-qPCR positivity rather than to estimate viral load. Instead, a fixed volume of 5 µL of extracted RNA was used per reaction, thereby standardising template input volume across samples.

RT-qPCR reactions were prepared according to the manufacturer’s instructions, using 15 µL of reaction mixture and 5 µL of extracted RNA per well. A CDV-positive control and a no-template negative control were included in each run, and an internal extraction control was used to monitor extraction efficiency and potential RT-qPCR inhibition. Reactions were loaded into a 96-well plate, sealed with optical adhesive film, briefly centrifuged to eliminate air bubbles, and processed on a QuantStudio™ 5 Real-Time PCR System (Thermo Fisher Scientific™, Waltham, MA, USA), using the thermal cycling conditions recommended by the manufacturer (Table 1).

For final validation of the analytical run, the following criteria had to be met:

• Amplification of the CDV RT-qPCR-positive control;
• No amplification of the no-template negative control;
• Adequate amplification of the internal extraction control, according to the manufacturer’s validation criteria.

Only results meeting these criteria were considered. Samples were classified as CDV RT-qPCR-positive when true target amplification was detected in the FAM™ channel, indicated by a typical sigmoidal amplification curve with a Cq value < 40. Samples were classified as CDV RT-qPCR-negative when no target amplification was detected in a valid reaction with adequate internal extraction control amplification. Samples were considered invalid when no CDV target amplification was detected and the internal extraction control failed to amplify and were therefore excluded from the study. In strongly positive samples, suppression or absence of the internal extraction control signal was not considered invalid when robust CDV target amplification was present, in accordance with the manufacturer’s interpretation criteria.

### 2.3. Statistical Analysis

Data were extracted from Sislab^®^ (Glintt, Global Intelligent Technologies, Lisbon, Portugal) and organised in Microsoft Excel^®^ (Microsoft, Redmond, WA, USA). Data integrity and completeness were verified prior to analysis. All statistical procedures were conducted using JMP^®^ v18.0.0 (SAS Institute, Cary, NC, USA) and DATAtab^®^ (numiqo e.U., Graz, Austria, 2026).

The primary outcome was the CDV RT-qPCR result, classified as positive or negative for CDV RNA. Categorical variables were summarised as absolute and relative frequencies, whereas continuous variables were described using mean, standard deviation, median, and range or interquartile range, as appropriate. Overall and annual CDV RT-qPCR positivity were calculated as the proportion of CDV RT-qPCR-positive samples among all valid samples tested, with annual estimates graphically represented using 95% confidence intervals (CI).

Associations between CDV RT-qPCR result and categorical variables, including sex, breed group, specimen type, and Nomenclature of Territorial Units for Statistics level 2 (NUTS 2) region, were assessed using Pearson’s chi-square test of independence. Fisher’s exact test or exact alternatives were considered when expected frequencies were low. Normality of continuous variables was assessed by graphical inspection and formal normality tests. As normality assumptions were not met, age was compared between RT-qPCR-positive and RT-qPCR-negative dogs using the Wilcoxon rank-sum test. As a secondary analysis, age was also categorised into four clinically relevant groups (<2 months, 2–6 months, 6–24 months, and >24 months), and its association with CDV RT-qPCR result was assessed using Pearson’s chi-square test or Fisher’s exact test, as appropriate.

Temporal trends in CDV RT-qPCR positivity were evaluated using segmented binomial regression models. Annual CDV RT-qPCR positivity was calculated as the proportion of positive samples among all tested samples for each year. A generalised linear model with binomial distribution and logit link was initially fitted using year as a continuous predictor. Segmented regression analysis was subsequently applied to identify potential breakpoints corresponding to significant changes in temporal trends. The breakpoint was estimated automatically by the model, and separate slopes were calculated before and after the identified breakpoint. Confidence intervals (95% CI) for annual positivity estimates were calculated.

All tests were two-sided, and statistical significance was set at *p* < 0.05. Results were interpreted considering the retrospective, laboratory-based, and submission-driven nature of the dataset, particularly for categories with small sample sizes or sparse expected frequencies.

## 3. Results

### 3.1. Descriptive Data

A total of 637 diagnostic samples from dogs with clinical suspicion of canine distemper were included in the study. Overall, 215 samples were CDV RT-qPCR-positive, yielding an overall positivity of 33.8% (95% CI: 30.2–37.5%).

### 3.2. Sex

Sex was recorded for all 637 dogs, including 300 females (47.1%) and 337 males (52.9%). CDV RT-qPCR positivity was similar in females and males, 32.7% and 34.7%, respectively. No significant association was observed between sex and CDV RT-qPCR result (χ^2^ = 0.299, *df* = 1, *p* = 0.585; Pearson’s chi-squared test), suggesting that sex was not associated with CDV molecular positivity in this dataset.

### 3.3. Age

Age information was available for 536/637 dogs (84.1%). As age (in months) was not normally distributed, it was summarised primarily using the median and range. The recorded age ranged from <1 to 204 months old (17 years old), with a median age of 8 months, confirming the predominance of young dogs among submitted cases. Age distribution was comparable between CDV RT-qPCR-positive and RT-qPCR-negative dogs, with median ages of 8 and 8.5 months, respectively. No statistically significant difference in age was observed between CDV RT-qPCR-positive and CDV RT-qPCR-negative dogs (Mann–Whitney W = 30798; *p* = 0.762). In the secondary categorical analysis, no significant association was observed between age group and CDV RT-qPCR positivity (χ^2^ = 1.44, *df* = 3, *p* = 0.695). These findings indicate that age was not significantly associated with CDV molecular positivity within this clinically suspected, submission-driven diagnostic population, but they should not be interpreted as excluding age-related susceptibility in the general dog population.

### 3.4. Breed

Breed information was available for all 637 submissions. Mixed-breed dogs were the predominant category, accounting for 456/637 cases (71.6%), whereas purebred dogs represented 181/637 submissions (28.4%).

A significant association was observed between breed group and CDV RT-qPCR result, with mixed-breed dogs showing a substantially higher proportion of positive results compared with other breeds (193/456, 42.3% vs. 22/181, 12.1%; χ^2^(1) = 52.75, *p* < 0.001; Pearson’s chi-squared test). This association should be interpreted as an unadjusted association with breed group, likely confounded by management, origin, vaccination access, and exposure history.

### 3.5. Specimen Type

The submitted biological specimens comprised EDTA-anticoagulated whole blood, conjunctival swabs, CSF, and rectal swabs. EDTA-anticoagulated whole blood was the predominant specimen type, accounting for 527/637 submissions (82.7%), followed by conjunctival swabs (59/637; 9.3%), CSF (43/637; 6.8%), and rectal swabs (8/637; 1.2%).

CDV RT-qPCR positivity differed significantly according to specimen type (*p* < 0.001; Fisher’s exact test). Positivity was highest in EDTA-anticoagulated whole blood (196/527; 37.2%) and conjunctival swabs (17/59; 28.8%), whereas lower positivity was observed in CSF (2/43; 4.6%) and rectal swabs (0/8; 0.0%). Because specimen types were unevenly represented, these results should not be interpreted as estimates of specimen-specific diagnostic sensitivity, specificity, or comparative diagnostic performance.

### 3.6. Geographical Distribution of CDV-Positive Animals

Submissions were geographically unevenly distributed across NUTS 2 regions, with the North region accounting for 506/637 submissions (79.4%). In this region, 171/506 submissions were CDV RT-qPCR-positive, corresponding to a positivity of 33.8%. Submissions from the autonomous insular regions were uncommon, accounting for 11/637 diagnostic submissions (1.7%). These included eight submissions from the Autonomous Region of Madeira, of which two were CDV RT-qPCR-positive (2/8; 25.0%), and three submissions from the Autonomous Region of the Azores, none of which were positive (0/3; 0.0%). No statistically significant association was detected between the NUTS 2 region and CDV RT-qPCR status (*p* = 0.441; Fisher’s exact test). The geographical distribution of diagnostic samples by mainland district is shown in Figure 1. District-level data, including the number of submitted samples, the number of CDV RT-qPCR-positive samples, positivity estimates and 95% confidence intervals, are provided in Table S1.

### 3.7. Temporal Distribution

Annual CDV RT-qPCR positivity remained comparatively high between 2013 and 2020, ranging from 23.8% to 54.5%. From 2021 onwards, a marked reduction in positivity was observed, with annual positivity decreasing to 10.0% in 2021 and remaining absent or very low between 2022 and 2025. Specifically, no positive samples were recorded in 2022, 2023, or 2025, while one positive sample was recorded in 2024, corresponding to an annual positivity of 2.5%.

**Figure 2:**
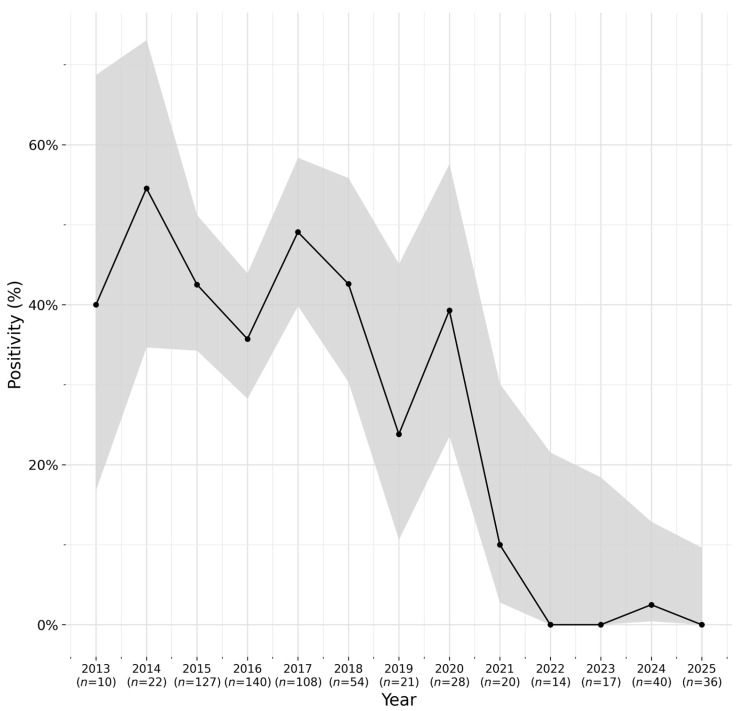
Temporal evolution of canine distemper virus (CDV) positivity by reverse transcription real-time PCR (RT-qPCR) in a qualitative approach among diagnostic samples from clinically suspected dogs in Portugal, 2013–2025. Annual positivity was calculated as the proportion of CDV RT-qPCR-positive samples among all valid submissions received in each calendar year. The black line and points represent annual positivity estimates, and the shaded area represents the 95% confidence interval. The annual number of samples is shown below each year (*n*).

Figure 2 shows the temporal evolution of CDV RT-qPCR positivity among diagnostic samples from clinically suspected dogs in Portugal, 2013–2025. Segmented binomial regression identified a breakpoint at approximately 2020. Before this point, no significant temporal trend in CDV RT-qPCR detection was observed (slope = −0.04; 95% CI: −0.21–0.13). From 2020 onwards, CDV RT-qPCR positivity showed a significant decreasing trend (slope = −1.19; 95% CI: −2.02 to −0.37).

**Figure 3:**
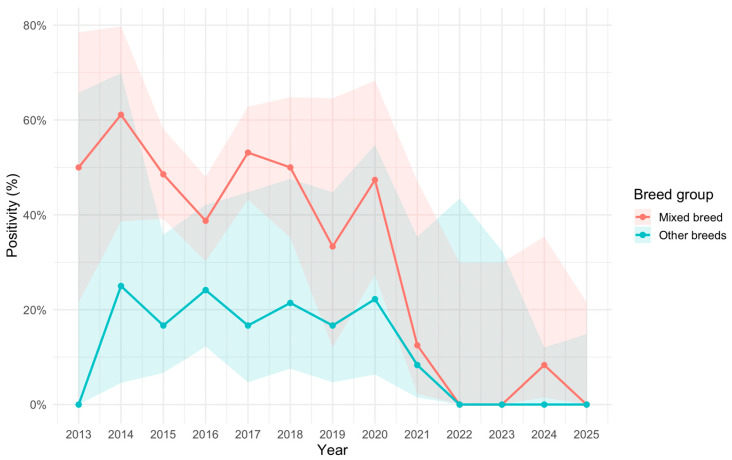
Temporal trends in canine distemper virus (CDV) RT-qPCR positivity according to breed group (“Mixed breed” and “Other breeds”) between 2013 and 2025. Points represent the annual positivity proportion, lines connect yearly estimates, and shaded areas indicate 95% Wilson confidence intervals.

Although mixed-breed dogs generally showed higher CDV RT-qPCR-positivity than other breeds, a marked decline in positivity was observed in both breed groups after 2020 (Figure 3).

## 4. Discussion

This retrospective molecular study provides a laboratory-based assessment of CDV detection in dogs with clinical suspicion of canine distemper in Portugal. Among 637 valid diagnostic samples, 215 were CDV RT-qPCR-positive, corresponding to an overall positivity of 33.8%. This finding confirms that CDV remained a clinically relevant and molecularly confirmed cause of compatible disease among the submitted population. However, the principal epidemiological observation was the marked temporal redistribution of positive results. CDV-positive submissions were concentrated in the earlier years of the study period, whereas a pronounced reduction in RT-qPCR positivity was observed after 2020, with very low or absent annual positivity between 2021 and 2025. This decline should be interpreted as reduced molecular detection among submitted clinically suspected dogs, rather than as evidence of national elimination of CDV or as a measured reduction in population-level incidence.

The overall positivity observed in this Portuguese diagnostic series is higher than that reported in some broader passive-surveillance studies, but lower than estimates generated during active outbreak investigations. The Southern Italy diagnostic series remains a useful comparator because it also relied on passive diagnostic submissions. In that study, Alfano et al. [31] detected CDV RNA in 6.8% of autochthonous and imported dogs investigated between 2014 and 2021, with no positive dogs reported in 2020–2021. By contrast, outbreak-based studies naturally produce higher positivity estimates because the denominator is restricted to clinically compatible animals sampled during periods of active transmission. For example, the 2019 Galápagos outbreak involved 125 dogs with clinical signs compatible with CDV and confirmed active circulation in a highly susceptible island dog population [32]. Therefore, the present estimate should not be interpreted as population prevalence. It reflects a diagnostic denominator shaped by clinical suspicion, veterinary access, sample selection, owner compliance, and submission practices.

The post-2020 decline is likely to be multifactorial. A genuine reduction in domestic-dog CDV circulation is biologically plausible, particularly if earlier transmission reduced the susceptible pool through mortality, naturally acquired immunity, and subsequent improvements in preventive management. CDV is an acute, highly contagious morbillivirus, and sustained transmission requires the continuous availability of susceptible hosts. Phylodynamic analyses have shown that CDV temporal patterns are influenced by viral dispersal, population susceptibility, and vaccination [33]. Accordingly, the high positivity observed during the earlier years of the present study may have reflected a period of increased susceptible-host availability, whereas the subsequent decline may partly represent contraction of that susceptible population.

Vaccination is central to this interpretation. Current WSAVA guidelines classify CDV vaccination as a core canine vaccine and recommend repeated puppy vaccination until at least 16 weeks of age to overcome maternally derived antibody interference, followed by appropriate booster strategies [8]. This is particularly relevant because the present study focused on dogs reported as unvaccinated, a group expected to be enriched for susceptibility. Portuguese shelter data also suggest that routine prophylactic measures, including vaccination protocols, are widely implemented in many municipal and non-profit animal shelters, although heterogeneity remains between institutions [34]. Improved vaccination practices in shelters, rescue networks, or owned-dog populations could therefore have contributed to a reduction in clinically apparent CDV, although this explanation remains hypothetical because national longitudinal data on CDV-specific vaccine coverage in Portuguese dogs were not available. Increased vaccine uptake or improved preventive healthcare may have contributed to the observed decline, but this remains inferential because the present dataset did not include longitudinal data on CDV vaccine coverage, owner behaviour or preventive healthcare compliance in Portuguese dogs.

A second, and probably equally important, explanation is a change in the submitted diagnostic population after 2020. Passive laboratory surveillance is intrinsically dependent on owner behaviour, veterinary access, clinical suspicion, sample selection, and submission practices. The COVID-19 pandemic substantially altered veterinary healthcare delivery and owner decision-making in several settings. Owczarczak–Garstecka et al. [35] showed that the pandemic affected owners’ veterinary healthcare-seeking behaviour for dogs, including access to care, treatment provision, and decisions regarding non-urgent or preventative veterinary visits. These effects are directly relevant to the present study because CDV diagnosis depends on a chain of events: the owner must seek veterinary care, the clinician must suspect distemper, an appropriate sample must be collected, and the sample must be submitted for molecular testing. Any disruption at one or more of these levels may reduce confirmed laboratory positivity, even if viral circulation has not disappeared. The decline should therefore be framed as a reduction in RT-qPCR positivity among submitted suspected cases, not as a measured decline in population-level incidence. The latter requires a defined population at risk, systematic case ascertainment, and stable diagnostic effort over time. Reduced contact rates among dogs during periods of social restriction may also have contributed to lower transmission risk. However, the present dataset does not include direct information on dog movement, owner behaviour, contact networks, or vaccine uptake. These mechanisms should therefore be regarded as plausible explanatory hypotheses rather than demonstrated causes of the observed decline. The available data do not allow the relative contribution of these mechanisms to be separated.

The influence of specimen type is also relevant to the temporal interpretation. In this study, CDV RT-qPCR positivity differed significantly according to specimen type, with the highest positivity observed in EDTA-anticoagulated whole blood and conjunctival swabs, and lower positivity in cerebrospinal fluid and rectal swabs. This pattern is consistent with the stage-dependent pathogenesis of canine distemper. During acute systemic infection, viral RNA is more likely to be detected in blood and mucosal secretions, whereas in chronic or delayed neurological disease, peripheral viraemia may have declined, and molecular detection from blood or conjunctival specimens may be less reliable. Previous studies have demonstrated the diagnostic value of RT-PCR or RT-qPCR in different clinical specimens, including whole blood, mucosal samples, and cerebrospinal fluid, but diagnostic sensitivity depends strongly on disease stage, clinical syndrome, and sample selection [22,23,36]. Nevertheless, because specimen types were not collected in parallel from the same animals and were highly unevenly represented, these findings should not be interpreted as demonstrating superior diagnostic sensitivity or specificity of EDTA-anticoagulated whole blood.

The absence of significant associations with sex, age, and NUTS 2 region should be interpreted within the constraints of a retrospective submission-driven dataset. Sex was not associated with RT-qPCR positivity, which is consistent with CDV risk being driven primarily by immunity, vaccination status, exposure, and management rather than intrinsic sex-related susceptibility [1,5,6,7]. Although young dogs predominated among submissions, age was not significantly associated with positivity when analysed either as a continuous variable or using clinically relevant age categories. This may reflect the fact that the study population was already restricted to clinically suspected and reportedly unvaccinated dogs, reducing the ability to identify an independent age effect within a high-risk diagnostic subset. Puppies and young dogs are classically considered at higher risk of severe distemper because of incomplete vaccination, maternally derived antibody interference, and immunological immaturity. WSAVA guidelines specifically address this risk by recommending repeated core vaccination through the puppy period, with final puppy doses at or beyond 16 weeks of age [8]. In addition, age was missing for 15.9% of dogs, and the age distribution was skewed towards younger animals, which may have limited statistical power.

Breed-related findings should be interpreted cautiously. Breed was analysed as a broad binary variable, comparing mixed-breed dogs with all other reported breeds combined, because most individual breeds were represented by small and uneven numbers of submissions. Therefore, the higher crude RT-qPCR positivity observed among mixed-breed dogs should be regarded as an unadjusted exploratory finding, not as evidence of breed-associated or genetic susceptibility. Likewise, the absence of detectable differences among individual breeds cannot exclude true breed-level effects, as the dataset lacked sufficient statistical power for such comparisons.

Geographical findings were similarly constrained by the structure of the dataset. Most submissions originated from northern Portugal, reflecting the catchment area and referral structure of the diagnostic laboratory. The absence of a significant association between NUTS 2 region and RT-qPCR status does not exclude spatial heterogeneity in CDV circulation. Passive laboratory datasets are vulnerable to referral bias, unequal veterinary access, and regional differences in clinician awareness or diagnostic habits. Consequently, the present study provides valuable multicentre laboratory evidence from Portugal, but it should not be interpreted as a fully representative national prevalence survey or as evidence of homogeneous regional CDV circulation.

From the perspective of domestic dog–wildlife interface epidemiology, the decline in domestic-dog RT-qPCR positivity should not be equated with the disappearance of CDV from Portuguese or Iberian ecosystems. CDV is a multi-host pathogen with a broad host range, including domestic dogs, wild canids, mustelids, procyonids, felids, and other susceptible mammals [14,37,38]. Portuguese and Iberian data have documented exposure or molecular evidence of CDV infection in free-ranging carnivores, including wolves, red foxes, and Iberian lynxes [2,12,13,29]. More recent European studies have confirmed ongoing CDV circulation in wildlife systems, including red foxes, mustelids, and other wild carnivores [7,9,10,11,39,40,41]. Therefore, low apparent positivity in a passive domestic-dog diagnostic system may coexist with continued viral circulation in wildlife reservoirs or multi-host metareservoirs. These findings are therefore relevant to domestic dog–wildlife interface surveillance, particularly because CDV monitoring requires consideration of domestic animal health, wildlife conservation, vaccination practices, and human-mediated animal management.

This has direct implications for interpreting the post-2020 decline. Domestic dogs may act as sources of infection for wildlife, particularly when unvaccinated, free-roaming, hunting, or shelter-associated populations overlap with susceptible wild carnivores. Conversely, wildlife may maintain or amplify CDV under specific ecological conditions and may contribute to episodic spillback into domestic dogs. Rural and peri-urban landscapes in Portugal provide opportunities for contact between domestic dogs, red foxes, wolves, mustelids, and other carnivores. The observed reduction in canine diagnostic positivity should therefore be viewed as encouraging, but not as definitive evidence of ecological control. Continued surveillance at the domestic dog–wildlife interface remains warranted.

Viral evolution and diagnostic inclusivity are also relevant. CDV displays marked genetic diversity, particularly in the haemagglutinin gene, which is important for receptor interaction, host adaptation, and phylogenetic classification [15,42]. Although modern RT-qPCR assays usually target conserved regions, genetic divergence can theoretically affect assay sensitivity if mutations occur in primer or probe binding sites [24,25].

Several limitations must be considered for the present study. First, this was a retrospective, laboratory-based, and submission-driven study, and the denominator consisted of dogs submitted for diagnostic testing rather than a defined population at risk. Therefore, incidence, prevalence, and transmission rate cannot be inferred. Second, annual submission numbers and clinical suspicion patterns may have varied over time, particularly after 2020, potentially influencing observed positivity. Third, vaccination status was based on information available in laboratory requisition forms and may have been incomplete or inconsistently recorded. Fourth, clinical information was limited, preventing robust stratification by syndrome, disease stage, disease duration, or previous treatment. Fifth, Cq values were not analysed, and viral load could not be used to differentiate acute high-burden infections from low-copy or late-stage detections. Finally, positive samples were not sequenced, preventing assessment of circulating lineages, possible lineage replacement, domestic–wildlife transmission links, or assay inclusivity across genetically divergent strains.

Taken together, the most defensible interpretation is that the post-2020 decline reflects a combination of epidemiological and surveillance-related processes. A true reduction in domestic dog CDV circulation is plausible, particularly if earlier transmission reduced the susceptible pool and vaccination or preventive practices improved. However, the decline may also have been amplified by changes in veterinary healthcare access, owner behaviour, diagnostic suspicion, clinical stage at sampling, specimen selection, annual denominators, and the inherent limitations of passive surveillance. The available data do not allow these mechanisms to be separated conclusively.

### Recommendations

Future studies should therefore integrate domestic dog and wildlife surveillance, particularly at domestic dog–wildlife interfaces. Continued vaccination of domestic dogs remains essential, particularly in puppies, shelters, rescue populations, hunting dogs, and free-roaming dogs. This is particularly important because apparent reductions in CDV incidence may lead to complacency and declining vaccine uptake, thereby allowing susceptible cohorts to accumulate and creating conditions for future re-emergence. Historical patterns of canine distemper control indicate that periods of reduced case detection should reinforce, rather than weaken, vaccination efforts. At the same time, molecular surveillance should include wildlife rehabilitation centres, necropsy submissions, wild carnivore mortality events, and domestic dogs from interface areas. Such an approach would allow detection of re-emergence, lineage shifts, and possible bidirectional transmission between domestic and wild hosts. Given the continuing detection of CDV in European wildlife, the absence of recent positives in submitted Portuguese dogs should be viewed as encouraging but not definitive evidence of control.

## 5. Conclusions

This study documents a marked decline in CDV RT-qPCR positivity in Portuguese diagnostic samples after 2020, but the underlying mechanism is likely multifactorial. The most plausible contributors are changes in submission patterns and veterinary access around the COVID-19 period, compounded by small annual post-2020 denominators, although reduced contact rates among dogs, improved vaccine awareness, increased population immunity, and reduced susceptible-host availability may also have contributed. Because CDV continues to circulate at the domestic dog–wildlife interface in the Iberian Peninsula and wider Europe, low positivity in submitted dogs should be interpreted as reduced detection within a passive diagnostic population rather than as evidence of elimination. Continued vaccination, appropriate specimen selection, and strengthened molecular and epidemiological surveillance at domestic dog–wildlife interfaces remain essential.

## Figures and Tables

**Table 1:** Thermal cycling conditions used for the detection of canine distemper virus.

Cycles	Temperature	Time	Notes
1	50 °C	20 min	Reverse transcription
1	95 °C	2 min	Polymerase activation
40	95 °C	5 s	Denaturation
	60 °C	30 s	Annealing/Extension

## Data Availability

The data presented in this study are available upon request from the corresponding authors.

## Supplementary Materials

The following supporting information can be downloaded at: https://www.mdpi.com/article/10.3390/v18070734/s1, Table S1. Distribution of canine distemper virus real time-qualitative PCR diagnostic submissions from clinically suspected dogs in Portugal by mainland districts or Autonomous Regions.

## Abbreviations

The following abbreviations are used in this manuscript:

CDV	Canine distemper virus
CSF	Cerebrospinal fluid
EDTA	Ethylenediaminetetraacetic acid
IEC	Internal extraction control
IQR	Interquartile range
NUTS	Nomenclature of Territorial Units for Statistics
RT-qPCR	Reverse transcription quantitative polymerase chain reaction
WSAVA	World Small Animal Veterinary Association
