# Supplementary material for: Molecular Detection of Canine Distemper Virus in Portugal: What Explains the Post-2020 Decline? A Retrospective RT-qPCR Study
Source: Viruses. 2026 Jul 2;18(7):734. doi: 10.3390/v18070734 (PMC13431585; doi:10.3390/v18070734)
Supplement: Supplementary file 1 [file viruses-18-00734-s001.zip › viruses-4374967-supplementary.pdf]

Table S1. Distribution of canine distemper virus real time-qualitative PCR diagnostic submissions from clinically suspected dogs in Portugal by mainland districts or Autonomous Regions.

District	Samples (*n*)	Positive (*n*)	Positivity (%)	95% CI
Aveiro	50	13	26.0	15.9–39.6
Braga	29	11	37.9	22.7–56.0
Bragança	13	5	38.5	17.7–64.5
Castelo Branco	3	0	0.0	0.0–56.2
Coimbra	23	13	56.5	36.8–74.4
Évora	2	0	0.0	0.0–65.8
Faro	17	5	29.4	13.3–53.1
Guarda	2	0	0.0	0.0–65.8
Leiria	7	1	14.3	2.6–51.3
Lisboa	9	4	44.4	18.9–73.3
Porto	344	95	27.6	23.1–32.6
Santarém	11	5	45.5	21.3–72.0
Setúbal	9	2	22.2	6.3–54.7
Viana do Castelo	2	0	0.0	0.0–65.8
Vila Real	102	58	56.9	47.2–66.1
Viseu	3	1	33.3	6.2–79.2
Autonomous Regions	11	2	18.2	5.1–47.7
**Total**	**637**	**215**	**33.8**	**30.1–37.6**

CI: confidence interval.
